# Might Thyroid Function in Patients with Turner Syndrome Have a Significant Impact on Their Muscle Strength?

**DOI:** 10.3390/ijms26083679

**Published:** 2025-04-13

**Authors:** Mariola Krzyscin, Elżbieta Sowińska-Przepiera, Žana Bumbulienė, Anhelli Syrenicz

**Affiliations:** 1 Pediatric, Adolescent Gynecology Clinic, Department of Gynecology, Endocrinology and Gynecological Oncology, Pomeranian Medical University in Szczecin, Unii Lubelskiej 1, 71-252 Szczecin, Poland; 2 Department of Endocrinology, Metabolic and Internal Diseases, Pomeranian Medical University in Szczecin, Unii Lubelskiej 1, 71-252 Szczecin, Poland; 3 Clinic of Obstetrics and Gynecology, Institute of Clinical Medicine, Faculty of Medicine, Vilnius University, LT-08661 Vilnius, Lithuania

**Keywords:** Turner syndrome, muscle strength, autoimmune thyroiditis, thyroid

## Abstract

Turner syndrome (TS) is a genetic disorder caused by abnormalities in one of the X chromosomes. Individuals with TS have a higher incidence of autoimmune thyroid disorders, particularly Hashimoto’s disease, leading to thyroid dysfunction, most commonly hypothyroidism. Hormonal imbalance, growth hormone deficiency, and reduced physical activity contribute to muscle weakness in TS patients, and thyroid dysfunction can exacerbate these effects. The purpose of this study was to evaluate whether thyroid factors affect muscle strength in female patients with TS. The study included 70 women with TS and 88 age- and weight-matched controls. TS diagnoses were genetically confirmed (mosaic karyotypes: *n* = 20; monosomy X: *n* = 37; structural abnormalities: *n* = 7). The main criterion for exclusion from the study was unbalanced thyroid function. Serum levels of thyroid-stimulating hormone (TSH), free thyroxine (fT4), free triiodothyronine (fT3), and thyroid antibodies (anti-thyroid peroxidase antibodies (aTPO), anti-thyroglobulin antibodies (aTG)) were measured, and muscle strength was assessed using hand-held dynamometry. In TS patients, higher TSH levels were positively correlated, and higher fT4 levels were negatively correlated with muscle strength. No such correlations were found in controls. Thyroid compensation may impact musculoskeletal health in TS. Lower-normal TSH levels are associated with reduced muscle strength, and autoimmune thyroid changes like aTPO and aTG may contribute to muscle deterioration. Further research is needed to confirm these findings.

## 1. Introduction

Turner syndrome (TS) is a genetic disorder characterized by the presence of quantitative and/or structural abnormalities in one of the two X chromosomes, often accompanied by mosaicism. The prevalence of TS is estimated to be between 1 in 2000 and 1 in 2500 live female births [1]. Globally, approximately 1.5 million women are affected by TS [1]. Recognized X chromosome aberrations in TS include X monosomy (45,X) and various forms of mosaicism (50–70%), such as 45X/46,XX, 45,X/46XY, 45,X/46,XX/47,XXX, 45,X/46X,i(Xq), and 45,X/46,Xdel(Xp). Additionally, structural abnormalities of the X chromosome include total or partial deletion of the short arm (*46,Xdel(Xp)*), isochromosome of the long arm (*46,X,i(Xq)*), ring chromosome (*46,X,r(X)*), and marker chromosome *(46,X+m)* [1,2].

People with TS experience significantly increased morbidity. The syndrome is often accompanied by osteoporosis, visual defects, hearing loss, cardiovascular disease (hypertension, increased risk of ischemic heart disease and congenital heart defects: bicuspid aortic valve and coarctation of the aorta), and renal anomalies (horseshoe kidney, double pelvis, or double kidney) [3,4,5,6]. In addition, individuals with TS in both pediatric and adult populations have a higher prevalence of several autoimmune disorders, including autoimmune thyroid disease (ATD), celiac disease, inflammatory bowel disease, psoriasis, acquired vitiligo, and juvenile rheumatoid arthritis. Symptoms and clinical presentation can vary considerably from patient to patient depending on the type of X chromosome aberration [7,8,9]. Previous research has shown that haploinsufficiency of at least ten genes located on the X chromosome affects immune regulation by influencing self-protein exposure in the thymus and the escape of autoreactive T cells [9,10]. Findings by Bakalov et al. suggest that ovarian insufficiency-related factors may contribute to these autoimmune conditions [11].

ATDs are more common in individuals with TS compared to healthy peers, covering a range of phenotypes. These include Hashimoto’s thyroiditis (HT) and Graves’ disease (GD), with HT being the most prevalent autoimmune thyroid disease and GD causing hyperthyroidism [12,13,14]. Most cases of HT eventually lead to hypothyroidism, although patients may not show symptoms initially. The antibodies slowly damage the thyroid gland, impairing its ability to produce hormones. This is particularly important for high-risk groups, such as patients with TS. Since the process is gradual, patients with TS may not notice these changes early on [15,16].

Besides, patients with TS face skeletal and muscular challenges due to estrogen deficiency resulting from ovarian insufficiency, leading to reduced bone density [17,18]. While estrogen replacement therapy is commonly used, the effects of other endocrine factors such as growth hormone (GH) and thyroid function on bone health require further investigation [19,20]. TS individuals also experience muscle issues, including weakness, limited functionality, and a higher risk of metabolic disorders [21,22]. These muscle weaknesses are attributed to hormonal imbalances, reduced physical activity, and GH deficiency [23,24].

It is a well-known fact that both hyperthyroidism and hypothyroidism have a negative effect on muscle strength and performance. In the case of hyperthyroidism, there is an increased breakdown of muscle proteins and greater energy consumption by the muscles, which can lead to weakness. Patients then experience muscle pain and show reduced physical performance [25,26]. Individuals with hypothyroidism often experience decreased blood circulation to their muscles, leading to reduced muscle strength and slower reaction time. The most common complaints among hypothyroid patients are fatigue and muscle weakness [27,28]. Furthermore, studies show that even subclinical thyroid dysfunction, which does not yet cause clinical symptoms, can affect muscle strength [29,30].

The results of the above studies can only make a small contribution to our understanding of muscle health in TS, as most people at risk of TS-related weakness have thyroid hormone levels within the reference range. These women with TS generally undergo regular check-ups, and, if thyroid disorders are detected, they are quickly treated. Therefore, the question of whether the relationship between thyroid function and muscle metabolism also exists in the euthyroid range in this patient group is clinically important. Recently, an increasing number of cohort studies have emerged to assess the relationship between thyroid function and muscle metabolism in the euthyroid range [31,32,33,34,35].

In this study, we attempted to evaluate whether the levels of thyroid stimulating hormone (TSH), free thyroxine (fT4), and free triiodothyronine (fT3), as well as serum levels of anti-thyroglobulin antibodies (aTG) and anti-thyroid peroxidase antibodies (aTPO), affect hand grip strength (HGS) in patients with thyroid-corrected TS.

## 2. Results

Demographic and anthropometric analysis between TS patients and healthy women showed no differences in all parameters except height (Table 1). It turned out that patients with TS had a significantly lower height compared to healthy premenopausal women.

The analysis revealed statistically significant differences between the compared groups in TSH, aTPO, aTG, and hand grip mean (HGM)—an average of HGS from three measurements of both hands (Table 2). Although patients with unbalanced thyroid function were initially excluded, it still turned out that patients in the study group had significantly higher TSH values, significantly higher aTPO and aTG levels, and significantly lower HGM compared to patients in the control group. However, it should be noted that the effects observed for TSH, aTPO, and aTG were weak (Cohen’s *d* < 0.50), whereas the effect observed for HGM was strong (Cohen’s *d* > 0.80).

Patients with TS had a higher prevalence of antithyroid antibodies. The prevalence of aTG antibodies was significantly higher among patients with TS, while the prevalence of aTPO only showed a tendency to increase but did not reach the level of statistical significance (*p* > 0.05) (Table 3).

The analysis showed statistically significant differences in HGM levels between patients differentiated due to the presence of aTPO antibodies, and between patients differentiated due to the presence of aTG antibodies, both among patients with TS and in CGj. It turned out that in each group, those with aTPO or aTG antibodies were characterized by significantly lower muscle strength compared to patients without these antibodies. In addition, it is worth noting that the effects observed with aTG antibodies were strong (Cohen’s *d* > 0.80), while the effects observed with aTPO antibodies were moderate (Cohen’s *d* < 0.50 < 0.80) (Table 4).

The analysis showed that among those with TS, muscle strength increased with increasing TSH levels, while it decreased with increasing fT4 (Table 5). However, the correlations were weak. None of these findings were found in CG.

The next stage of the analysis was the verification of regression models checking to what extent individual parameters of thyroid function (TSH, fT3, fT4, fT3/fT4) explain muscle strength (HGM). Calculations were carried out separately among patients with TS and separately in CG. Before starting the analysis, the assumption of homoskedastic predictors was verified. Next, the assumption of non-collinearity of predictors was verified. It turned out that the variables FT3, FT4, and FT3/FT4 were characterized by a high degree of collinearity (VIF > 5.00), so it was decided to perform a hierarchical analysis using the backward elimination method. The results for the tested models are presented in Table 6.

It turned out that the final model obtained in the case of the control group was well suited to the data, explaining 6.8% of the variance of the dependent variable. A statistically significant predictor for muscle strength was the fT4 parameter, where a positive value of the Beta coefficient indicated that muscle strength increased with the increase in the level of the fT4 parameter, which was not found in patients with TS.

Next, we proceeded to analyze the prevalence of antithyroid antibodies (aTPO, aTG, or aTPO and aTG combined) according to genetic abnormalities, respectively: mosaic karyotype (*45,X/46,XX; 45,X/46,XX/47,XXX; or 45,X/47,XXX*), monosomy *45,X*, and others (isochromosome of the long arm of X (*46,X,i(Xq*)), ring chromosome (*46,X,r(X*))). (Table 7).

It turned out that patients with monosomy and isochromosome X, compared to mosaic patients, had significantly more often aTPO, aTG, and aTPO and aTG combined. In addition, isochromosome X patients were significantly more likely to have aTPO compared to monosomy patients. Moreover, it is noteworthy that all of the observed effects were found to be strong (Vc > 0.50).

However, the analysis showed no statistically significant difference between the different TS genotypes in terms of muscle strength (Table 8). This meant that individuals characterized by different genetic variants did not differ in terms of muscle strength.

## 3. Discussion

In this study, we analyzed thyroid function parameters, the presence of thyroid antibodies (aTPO, aTG), and their potential impact on muscle strength in individuals with TS who are thyroid-compensated and an age- and weight-matched control group of healthy, premenopausal women.

The *X* chromosome contains most immunity-related genes [36,37]. While men, with only one *X* chromosome, are more susceptible to *X*-linked immunodeficiencies, females typically have a more robust immune response due to two *X* chromosomes, though this increases their vulnerability to autoimmune diseases [38].

TS, characterized by the partial or complete absence of one *X* chromosome in phenotypic females, is a valuable model for studying *X* chromosome gene expression in immune function [37,38]. Genes such as *FOXP3* and *PTPN22* are believed to play a role in TS development [39]. Autoimmune disorders occur 2–3 times more frequently in women with TS than in the general population [1,40,41]. Several hypotheses, including skewed X inactivation and haploinsufficiency, link the *X* chromosome to this higher prevalence of autoimmune diseases, although the exact immunological mechanisms remain unclear. Preliminary studies suggest altered immunoglobulin levels and reduced T-lymphocyte subpopulations, with a lower CD4+/CD8+ T-lymphocyte ratio indicating potential immune response deficits in TS [41]. Su et al. identified differential methylation of *IL3RA* and *CSF2RA* genes on the *X* chromosome in women with TS, suggesting a potential link to autoimmune diseases like thyroiditis [42]. Despite some immunological abnormalities, patients with TS do not show an increased frequency of infections, except for recurrent otitis media. Additionally, many adults with TS exhibit low antibody titers after immunization, underscoring the need for further research into the clinical implications of these immune features [41].

Individuals with TS are at a higher risk of developing thyroid disorders, including autoimmune conditions such as HT, with subclinical hypothyroidism being particularly prevalent. Around 58% of women with TS experience this condition, which can manifest at any age [40,43,44].

The results of our study showed that patients with TS had higher absolute levels of aTPO and aTG antibodies compared to peers without TS. Additionally, there was a higher frequency of elevated aTG in TS patients, although this difference was borderline significant (*p* = 0.046). We also found that individuals with monosomy, isochromosome *X (46,X,i(Xq))*, or ring chromosome *X (46,X,r(X))* were more likely to have antithyroid antibodies than those with a mosaic karyotype. These findings confirm that certain genetic variants in TS are linked to a higher risk of autoimmune thyroid disorders.

Due to the increased incidence of autoimmune diseases in TS, current guidelines recommend thyroid function screening (TSH, fT4) from early childhood, with annual follow-up, but routine thyroid antibody testing is not advised [1]. Other common autoimmune diseases in TS include type 1 diabetes, celiac disease, alopecia areata, inflammatory bowel diseases, and skin conditions like dermatitis, eczema, and psoriasis [1,45]. Routine screening for celiac disease should begin in early childhood [1,45,46].

Studies suggest that GH treatment in GH-deficient patients, such as women with TS, can affect thyroid function [46]. GH is believed to regulate peripheral T4 metabolism by increasing the conversion of T4 to T3 [47], with changes observed in fT4 and fT3 levels. This effect likely involves deiodinase type 2, an enzyme crucial for converting T4 to the active thyroid hormone T3 in peripheral tissues, influencing thyroid hormone levels and metabolic processes [48].

One of the findings of our study was significantly higher TSH levels in the TS group compared to controls (Table 2). However, as our study was retrospective, we assessed thyroid parameters in both non-thyroid individuals and those with previously diagnosed and treated thyroid disease (17 of 77 patients with TS). These results align with other studies showing slightly higher TSH concentrations in individuals with TS [12].

Chronic thyroid disorders, such as autoimmune thyroiditis, can cause elevated TSH levels, typical for this patient group [49]. A transition from hyperthyroidism to hypothyroidism may also occur over time [25]. In the general population, thyroiditis is diagnosed based on clinical signs and lab tests, but in TS, thyroid function is routinely monitored during follow-up, allowing detection of even subclinical changes [1,49].

Many studies using muscle strength tests, such as the HGS test, have shown lower muscle strength in TS patients compared to age- and sex-matched controls [17,21,22]. Our study confirmed significantly weakened hand grip in patients with *X* chromosome loss.

Several studies have confirmed the relationship between thyroid function and grip strength. Both low and high TSH levels are associated with lower grip strength [27,28]. In healthy older adults, mild hypothyroidism or higher normal TSH values may lose relevance to muscle function with age [33,34]. For example, Netzer et al. found that treating subclinical hypothyroidism had no impact on muscle function, strength, or mass in individuals aged 65 and older [29].

The thyroid axis plays a key role in regulating muscle strength through various mechanisms. Studies suggest that due to the presence of TSH receptors in muscle cells, TSH may directly affect muscle metabolism, independent of thyroid hormones. This may include effects on muscle fiber differentiation and regulation of energy metabolism, which is crucial for maintaining muscle strength [32]. One study found that men with low TSH levels had a higher risk of low muscle strength compared to those with normal levels [50]. In women, menopause may affect the thyroid–muscle strength relationship, as rapid estrogen decline may diminish TSH’s impact on muscle metabolism [50]. In female TS patients, many other factors, such as bone mineralization, sex hormone deficiency, overt GH treatment, or nutritional deficiencies, may have an additional and even more significant effect on muscle strength and physical functioning [49]. Among our TS patients, higher TSH levels correlated positively with greater muscle strength, which was not observed among healthy women (Table 4). We observed a different relationship in the control group, where higher levels of fT4 significantly affected greater muscle strength (Table 6). Other parameters of thyroid function compensation had no significant effect on muscle function in either group. Regarding the presence of antithyroid antibodies in our study, we found that regardless of the X chromosome deficit, the presence of antibodies was associated with significantly lower muscle strength. The effect of decreased muscle strength was more strongly associated with the presence of aTG antibodies than with the presence of aTPO antibodies.

However, it should be emphasized that our pilot study has certain limitations. First of all, the cross-sectional nature of the study does not provide a basis for assessing the causality of the observations described. Secondly, the size of the study group, which is rather small, is not large enough to draw general conclusions. Thirdly, in our study group, there were patients with different karyotypes, which does not allow a direct comparison. Fourthly, we measured thyroid hormone levels and muscle strength indices at only one time point. Furthermore, the lack of analysis of other factors that may influence muscle strength, such as physical activity levels, diet, or other comorbidities, may limit the ability to fully interpret the results. Despite all limitations, this study adds to the limited literature available on the possible association between serum TSH levels and muscle strength measurements in a specific group of female patients.

## 4. Methods and Materials

### 4.1. Subjects

Our study, conducted from January 2021 to September 2024 at a university hospital’s referral center, involved female participants aged 20 to 44 years. This study included 70 patients diagnosed with TS and a control group of 88 women matched by age and weight. To qualify, participants needed to have genetic abnormalities confirming a TS diagnosis. The main criterion for exclusion from the study was unbalanced thyroid function, exceeding our laboratory’s reference values [0.27–4.84 μIU/mL]. Those with type 1 diabetes or major cardiovascular or renal defects were also excluded. Among the TS patients, two had celiac disease, and one had Sjogren’s syndrome. Only three participants with TS occasionally smoked cigarettes (less than once a month), and none reported alcohol abuse. All patients with TS were under constant endocrinological care and underwent regular thyroid function tests, at least once a year. Of the 70 patients in the study group, 17 (24.2%) were treated with l-thyroxine for hypothyroidism (doses ranging from 25 to 125 micrograms of l-thyroxine were used, depending on the clinical situation).

All TS patients had ceased growing and presented the following karyotypes: mosaicism *(45,X/46,XX; 45,X/46,XX/47,XXX; or 45,X/47,XXX)* (*n* = 20); monosomy *X (45,X)* (*n* = 37); and isochromosome of the long arm of *X (46,X,i(Xq))*, ring chromosome *(46,X,r(X))* (*n* = 7). No participants in the study group had any part of the *Y* chromosome detected. All women with TS had received recombinant human growth hormone (rhGH) therapy previously, with dosages adjusted to their clinical response (1.2–2 mg/m^2^/day). The average duration of GH therapy was 5.0 years, with treatments starting between the ages of 1.3 and 12.5 years. Estrogen–progesterone therapy was administered to 39 patients to trigger sexual maturation and to 13 patients for secondary ovarian failure, while 18 patients exhibited spontaneous sexual maturation. For those requiring puberty development, transdermal estrogen therapy began at an average age of 12.1 years (range 10.9–13.5 years) with an initial dose of 17β-estradiol at 0.00625 mg/day, gradually increasing to 0.05 mg/day. After an average of 2.4 years, transdermal therapy continued in a sequential regimen, alternating between 17β-estradiol alone for two weeks and 17β-estradiol plus norethisterone acetate for another two weeks at a dose of 0.17 mg/day. At the time of the study, all TS patients were receiving the specified estrogen–progesterone therapy. None of the control group participants used hormonal contraceptives and all had regular menstrual cycles, ensuring clear differentiation between the treatment and control groups for evaluating the impact of thyroid hormone levels on muscle parameters.

The CG consisted of 88 healthy premenopausal, Caucasian women, matched for age and weight, recruited from the Endocrinology Outpatient Clinic of the Pomeranian Medical University in Szczecin, women in whom endocrine disorders were excluded. None of these participants had received l-thyroxine therapy, estrogen, progesterone, or testosterone therapy, used contraceptives, smoked, abused alcohol, or had a known genetic disease.

Neither the study group nor the CG included patients actively receiving antithyroid drugs for hyperthyroidism.

All participants underwent comprehensive medical histories and physical examinations. Height was measured while standing using a digital telescopic wall-mounted stadiometer, and weight was recorded to the nearest 0.1 kg using an electronic scale.

### 4.2. Biochemical Analyses

Venous blood samples were collected from all patients in the morning while they were fasting. Patients who were on l-thyroxine therapy in the morning refrained from taking their medication until after the blood draw. These blood samples were analyzed in the hospital’s prospective laboratory, part of the core laboratory at Pomeranian Medical University in Szczecin. The levels of aTPO and aTG in the serum were measured by radioimmunoassay using kits from Brahms PCT immunoassay on a Cobas E801 analyser (Roche Diagnostics, Rotkreuz, Switzerland). Concentrations exceeding 60 IU/mL were considered elevated for both aTPO and aTG. The levels of fT3 and fT4 were measured by electrochemiluminescence using kits from Roche Diagnostics, Rotkreuz, Switzerland. Reference values for TSH were 0.27–4.84 μIU/mL; for fT4, 0.93–1.7 ng/dL; and for fT3, 1.8–4.6 pg/mL.

### 4.3. Muscle Function Assessment

HGS was evaluated using a SAEHAN 5030J1 hydraulic manual dynamometer, calibrated to a 40 kg load. This test measured the isometric strength of the hand and forearm. During the assessment, patients stood with the arm being tested bent at a 90-degree angle, keeping it close to their body. Each participant was instructed to grip the dynamometer bar with maximum effort three times, followed by a 30 s rest, alternating between their right and left hands. The average of the three readings from each hand was recorded as an average measurement of muscle strength—HGM, expressed in kilograms.

### 4.4. Statistical Analysis

Statistical analyses were conducted using IBM SPSS Statistics 30 software Version 28. The analyses included basic descriptive statistics and the Shapiro–Wilk test to assess the distribution of the variables. The Student’s *t*-test for independent samples was utilized to compare two groups with normally distributed quantitative variables, while the Mann–Whitney test was applied to compare two groups with quantitative variables that did not follow a normal distribution. Pearson’s r correlation analysis was used to examine relationships between normally distributed quantitative variables, and Spearman’s rho correlation analysis was used for quantitative variables that did not follow a normal distribution. A hierarchical back-elimination linear regression analysis was performed in order to verify the predictive strength of individual explanatory variables on the explanatory variable. The Kruskal–Wallis test was employed to compare more than two groups with unequal sample sizes in terms of quantitative variable levels. The significance level for these analyses was set at α = 0.05.

## 5. Conclusions

In conclusion, the results of our study suggest a somewhat different role for thyroid compensation in relation to measures of musculoskeletal health in the TS population. TSH values at the lower end of the reference range may be associated with lower muscular strength in patients with an X chromosome defect, and these patients would perhaps benefit from less intensive treatment for hypothyroid disorders. Regular monitoring of not only thyroid hormone levels but also muscle strength can help to better tailor therapy. Individualization of treatment is key, as patients with TS may show different responses to hormone therapy than healthy patients. In addition, chronic autoimmune thyroid lesions, such as the presence of aTPO and aTG, contribute to the deterioration of muscle function parameters independent of the X chromosome defect. Further prospective studies are needed to clarify the significance of our findings.

## Figures and Tables

**Table 1:** Demographic and anthropometric characteristics in patients with TS (TS) and control group (CG) (*n* = 158).

	TS (*n* = 70)		CG (*n* = 88)			
Characteristic	*M*	*SD*	*M*	*SD*	*t*	*p*
age [years]	33.04	10.29	32.17	9.90	0.54	0.589
height [cm]	154.04	5.35	167.66	6.21	−14.54	<0.001
weight [kg]	64.87	7.39	63.18	11.40	1.12	0.263

TS—patients with Turner syndrome; CG—control group; *n*—number of observations; *M*—mean; *SD*—standard deviation; *t*—value of the Student’s *t*-test statistic; *p*—statistical significance.

**Table 2:** Comparison of the patients with TS with the control group in terms of thyroid function parameters and muscle strength (*n* = 158).

		TS (*n* = 70)		CG (*n* = 88)				
Characteristics	References	*M*	*SD*	*M*	*SD*	*t*/*Z*	*p*	Cohen’s *d* η^2^
TSH [μIU/mL]	[0.27–4.84]	2.85	1.54	2.29	1.06	2.59	0.011	0.43
fT4 [ng/dL]	[0.93–1.77]	1.29	0.20	1.24	0.16	1.51	0.135	0.25
fT3 ^a^ [pg/mL]	[1.8–4.6]	3.22	0.63	3.64	2.88	−0.28	0.778	<0.01
fT3/fT4 ^a^		2.57	0.71	3.01	2.61	−1.03	0.303	<0.01
aTPO [IU/mL]	<60	224.39	312.87	118.95	175.93	2.52	0.013	0.43
aTG [IU/mL]	<60	260.28	320.23	162.74	227.16	2.15	0.033	0.36
HGM [kg]		19.07	4.54	27.78	4.10	−12.65	<0.001	2.03

TS—patients with Turner syndrome; CG—control group; *n*—number of observations; *M*—mean; *SD*—standard deviation; *t*—value of Student’s *t*-test statistic; *Z*—value of Mann–Whitney test statistic; *p*—statistical significance; Cohen’s *d*—effect strength ratio for Student’s *t*-test; η^2^—effect strength ratio for Mann–Whitney test; ^a^—Mann–Whitney test results are reported; TSH—thyroid-stimulating hormone; fT4—free thyroxine; fT3—free triiodothyronine; aTG—anti-thyroglobulin antibodies; aTPO—anti-thyroid peroxidase antibodies; HGM—hand grip mean.

**Table 3:** Comparison of the patients with TS with the control group in terms of the presence of aTPO.

Characteristic		TS (*n* = 70)		CG (*n* = 88)				
		*N*	%	*N*	%	χ^2^	*p*	ϕ
aTPO	No antibodies	33	48.6	56	63.6	3.61	0.075	0.15
	Antibodies present	37	51.4	32	36.4			
aTG	No antibodies	32	45.7	54	61.4	2.05	0.046	0.19
	Antibodies present	38	54.3	34	38.6			
aTPO + aTG	No antibodies	29	41.4	38	43.2	0.05	0.872	0.02
	Antibodies present	41	58,6	50	56,8			

TS—patients with Turner syndrome; CG—control group; *n*—number of observations; χ^2^—chi-square test result; *p*—statistical significance; ϕ—strength of effect index; aTG—anti-thyroglobulin antibodies; aTPO—anti-thyroid peroxidase antibodies; aTPO—anti-thyroid peroxidase antibodies.

**Table 4:** Comparison of subjects with aTPO and aTG antibodies present with those without aTPO and aTG antibodies present in terms of muscle strength separately in the patients with TS and separately in the control group (*n* = 158).

Group	Antibodies		HGM				
			*M*	*SD*	*t*	*p*	*d* Cohena
TS (*n* = 70)	aTPO	Antibodies present (*n* = 36)	17.54	4.13	−2.88	0.005	0.69
		No antibodies (*n* = 34)	20.51	4.49			
	aTG	Antibodies present (*n* = 35)	17.19	4.47	−3.78	<0.001	0.90
		No antibodies (*n* = 35)	20.95	3.82			
CG (*n* = 88)	aTPO	Antibodies present (*n* = 32)	26.84	4.34	−3.29	<0.001	0.66
		No antibodies (*n* = 56)	29.44	3.03			
	aTG	Antibodies present (*n* = 34)	25.59	2.90	8.56	<0.001	1.88
		No antibodies (*n* = 54)	31.26	3.22			

TS—patients with Turner syndrome, CG—control group; *n*—number of observations; *M*—mean; *SD*—standard deviation; *t*—value of Student’s *t*-test statistic; *p*—statistical significance; Cohen’s *d*—coefficient of strength of effect for Student’s *t*-test; aTG—anti-thyroglobulin antibodies; aTPO—anti-thyroid peroxidase antibodies.

**Table 5:** Correlation of thyroid parameters with muscle strength separately in the study group and separately in the control group (*n* = 139).

	HGM [kg]			
	TS (*n* = 70)		CG (*n* = 88)	
Variable	*r* Pearson’s/ *rho* Spearman’s	*p*	*r* Pearson’s/ *rho* Spearman’s	*p*
TSH [μIU/mL]	0.19	0.012	−0.05	0.176
fT4 [ng/dL]	−0.20	0.031	0.24	0.076
fT3 ^a^ [pg/mL]	0.10	0.403	0.14	0.209
fT3/fT4 ^a^	−0.05	0.709	−0.05	0.615

TS—patients with Turner syndrome; CG—control grou; HGM—hand grip mean; TSH—thyroid stimulating hormone; fT4—free thyroxine; fT3—free triiodothyronine; r—Pearson’s correlation coefficient; rho—Spearman’s rank correlation coefficient; *p*—*p*-value (significance level); ^a^—the results of Spearman’s rho correlation analysis are reported; *n*—number of observations.

**Table 6:** Results of hierarchical regression analyses to explain muscle strength on the basis of individual parameters of thyroid function (TSH, fT3, fT4), separately in TS patients and separately in the control group (*n* = 158).

Group	Predictor	*B*	*SE*	*Beta*	*t*	*p*	*F*	*p*	*R* ^2^ *_adj._*
TS (*n* = 70)	(Constant)	17.13	3.51		4.88	<0.001	0.31	0.578	<0.01
	fT4	1.51	2.69	0.07	0.56	0.578			
CG (*n* = 88)	(Constant)	18.81	3.34		5.62	<0.001	7.14	0.009	0.07
	fT4	7.10	2.66	0.28	2.67	0.009			

TS—patients with Turner syndrome; CG—control group; *B*—non-standardized regression coefficient; *SE*—standard error; *Beta*—standardized regression coefficient; *t*—the value of the test statistic of the *t*-test; *p*—statistical significance; *F*—value of the test analysis of variance statistics; *R^2^_adj_.*—the value of the adjusted R-squared statistic; fT4—free thyroxine; *n*—number of observations.

**Table 7:** Comparison of individuals with genetic variants of TS (mosaicism (*45,X/46,XX; 45,X/46,XX/47,XXX; or 45,X/47,XXX*) (*n* = 20); monosomy *X (45,X)* (*n* = 37); others (isochromosome of the long arm of *X (46,X,i(Xq)*), ring chromosome (*46,X,r(X)*)) in terms of the presence of aTPO and aTG (*n* = 70).

		Mosaics (*n* = 20)		45.X (*n* = 37)		Others (*n* = 13)				
Characteristic		*n*	%	*n*	%	*n*	%	χ^2^	*p*	Vc
aTPO	No antibodies	17	85.0	15	40.5	1	7.7	23.39	<0.001	0.58
	Antibodies present	3	15.0	22	59.5	12	92.3			
aTG	No antibodies	17	85.0	15	40.5	0	0.0	34.32	<0.001	0.70
	Antibodies present	3	15.0	22	59.5	13	100.0			
aTPO + aTG	No antibodies	15	75.0	11	29.7	0	0.0	30.73	<0.001	0.66
	Antibodies present	5	25.0	26	70,3	13	100,0			

TS—patients with Turner syndrome; *n*—number of observations; χ^2^—chi-square test result; *p*—statistical significance; Vc—strength of effect index; aTG—anti-thyroglobulin antibodies; aTPO—anti-thyroid peroxidase antibodies.

**Table 8:** Comparison of individuals with individual genetic variants in terms of muscle strength (*n* = 70), mosaicism *(45,X/46,XX; 45,X/46,XX/47,XXX; or 45,X/47,XXX)* (*n* = 20); monosomy *X (45,X)* (*n* = 37); and isochromosome of the long arm of *X (46,X,i(Xq))*, ring chromosome *(46,X,r(X))*.

Genetic Variants of TS	HGM				
	*M*	*SD*	H(2)	*p*	η^2^
mosaicism *(45,X/46,XX; 45,X/46,XX/47,XXX; or 45,X/47,XXX)* (*n* = 20)	19.09	4.91	0.03	0.985	<0.01
monosomy *X (45,X)* (*n* = 37)	19.00	4.61			
isochromosome of the long arm of X *(46,X,i(Xq*), ring chromosome *(46,X,r(X))* (*n* = 13)	19.25	4.08			

TS—patients with Turner syndrome; *n*—number of observations; *M*—mean; *SD*—standard deviation; H—value of test statistic; *p*—statistical significance; η^2^—strength of effect index; HGM—hand grip mean.

## Data Availability

Data are available upon special request by contacting the corresponding author (M.K.).

## References

[1] Gravholt C.H., Andersen N.H., Christin-Maitre S., Davis S.M., Duijnhouwer A., Gawlik A., Maciel-Guerra A.T., Gutmark-Little I., Fleischer K., Hong D. (2024). Clinical practice guidelines for the care of girls and women with Turner syndrome. Eur. J. Endocrinol..

[2] Queremel Milani D.A., Chauhan P.R. (2023). Genetics, Mosaicism. StatPearls.

[3] Lacka K. (2005). Turner Syndrome—Correlation between kariotype and fenotype. Endokrynol. Pol..

[4] Gravholt C. (2004). Epidemiological, endocrine and metabolic features in Turner Syndrome. Eur. J. Endocrinol..

[5] Aversa T., Gallizzi R., Salzano G., Zirilli G., De Luca F., Valenzise M. (2018). Atypical phenotypic aspects of autoimmune thyroid disorders in young patients with Turner Syndrome. Ital. J. Pediatr..

[6] Aversa T., Lombardo F., Valenzise M., Messina M., Sferlazzas C., Salzano G., De Luca F., Wasniewska M. (2015). Peculiarities of autoimmune thyroid diseases in children with Turner or Down syndrome: An overview. Ital. J. Pediatr..

[7] Gravholt C.H., Viuff M., Just J., Sandahl K., Brun S., van der Velden J., Andersen N.H., Skakkebaek A. (2023). The Changing Face of Turner Syndrome. Endocr. Rev..

[8] Berglund A., Stochholm K., Gravholt C.H. (2020). The epidemiology of sex chromosome abnormalities. Am. J. Med. Genet. C Semin. Med. Genet..

[9] Kanakatti Shankar R., Gravholt C.H., Backeljauw P.F. (2024). Evolution of Health Care in Turner Syndrome. Am. J. Med. Genet. C Semin. Med. Genet..

[10] De Marqui A. (2015). Turner Syndrome and genetic polymorphism: A systematic review. Rev. Paul. Pediatria.

[11] Bakalov V., Gutin L., Cheng C., Zhou J., Sheth P., Shah K., Arepalli S., Vanderhoof V., Nelson L.M., Bondy C.A. (2012). Autoimmune disorders in women with Turner Syndrome and women with karyotypically normal primary ovarian insufficiency. J. Autoimmun..

[12] Wasniewska M., Salerno M., Corrias A., Mazzanti L., Matarazzo P., Corica D., Aversa T., Messina M.F., De Luca F., Valenzise M. (2016). The evolution of thyroid function after presenting with Hashimoto Thyroiditis is different between initially Euthyroid girls with and those without Turner Syndrome. Horm. Res. Paediatr..

[13] El-Mansoury M., Bryman I., Berntorp K., Hanson C., Wilhelmsen L., Landin-Wilhelmsen K. (2005). Hypthyroidism is common in Turner Syndrome: Results of a five-year follow-up. J. Clin. Endorinol. Metab..

[14] Brix T., Hegedüs L. (2012). Twin studies as a model for exploring the aetiology of autoimmune thyroid disease. Clin. Endocrinol..

[15] Medeiros C.C., Marini S.H., Baptista M.T., Guerra G., Maciel-Guerra A.T. (2000). Turner’s syndrome and thyroid disease: A transverse study of pediatric patients in Brazil. J. Pediatr. Endocrinol. Metab..

[16] Caturegli P., De Remigis A., Rose N. (2014). Hashimoto thyroiditis: Clinical and diagnostic criteria. Autoimmun. Rev..

[17] Krzyścin M., Gruca-Stryjak K., Soszka-Przepiera E., Syrenicz I., Przepiera A., Cymbaluk-Płoska A., Bumbulienė Ž., Sowińska-Przepiera E. (2023). The Interplay between Muscular Grip Strength and Bone Mineral Density with Consideration of Metabolic and Endocrine Parameters in Individuals with Turner Syndrome. Biomedicines.

[18] Ikegawa K., Koga E., Itonaga T., Sakakibara H., Kawai M., Hasegawa Y. (2024). Factors associated with low bone mineral density in Turner syndrome: A multicenter prospective observational study. Endocr. J..

[19] Roy R., Hazra A., Ghosh S. (2023). An Observational Study on Response to Growth Hormone Therapy in Indian Patients of Short Stature with Special Emphasis on Biochemical Parameters and Bone Biomarkers. Indian J. Endocrinol. Metab..

[20] Szybiak W., Kujawa B., Miedziaszczyk M., Lacka K. (2023). Effect of Growth Hormone and Estrogen Replacement Therapy on Bone Mineral Density in Women with Turner Syndrome: A Meta-Analysis and Systematic Review. Pharmaceuticals.

[21] Soucek O., Lebl J., Matyskova J., Snajderova M., Kolouskova S., Pruhova S., Hlavka Z., Sumnik Z. (2015). Muscle function in Turner syndrome: Normal force but decreased power. Clin. Endocrinol..

[22] Soucek O., Matyskova J., Anliker E., Toigo M., Hlavka Z., Lebl J., Sumnik Z. (2015). The muscle-bone interaction in Turner syndrome. Bone.

[23] Milde K., Tomaszewski P., Stupnicki R. (2013). Physical fitness of schoolgirls with Turner syndrome. Pediatr. Exerc. Sci..

[24] Samad N., Chiu W.L., Nguyen H.H., Lu Z.X., Zacharin M., Ebeling P.R., Teede H., Scott D., Milat F., Vincent A.J. (2024). Impaired Muscle Parameters in Individuals with Premature Ovarian Insufficiency: A Pilot Study. J. Endocr. Soc..

[25] Spira D., Buchmann N., Demuth I., Steinhagen-Thiessen E., Völzke H., Ittermann T. (2019). Association of Thyroid Function with Handgrip Strength: Data from the Study of Health in Pomerania and the Berlin Aging Study II. Thyroid.

[26] Erkol İnal E., Çarlı A.B., Çanak S., Aksu O., Köroğlu B.K., Savaş S. (2015). Effects of hyperthyroidism on hand grip strength and function. J. Rehabil. Res. Dev..

[27] Di Iorio A., Paganelli R., Abate M., Barassi G., Ireland A., Macchi C., Molino-Lova R., Cecchi F. (2021). Thyroid hormone signaling is associated with physical performance, muscle mass, and strength in a cohort of oldest-old: Results from the Mugello study. Geroscience.

[28] Hata S., Okada H., Minamida M., Hironaka J., Hasegawa Y., Kondo Y., Nakajima H., Kitagawa N., Okamura T., Hashimoto Y. (2024). Associations between thyroid hormones and appendicular skeletal muscle index, and hand grip strength in people with diabetes: The KAMOGAWA-A study. Diabetes Res. Clin. Pract..

[29] Netzer S., Chocano-Bedoya P., Feller M., Janett-Pellegri C., Wildisen L., Büchi A.E., Moutzouri E., Rodriguez E.G., Collet T.H., Poortvliet R.K.E. (2023). The effect of thyroid hormone therapy on muscle function, strength and mass in older adults with subclinical hypothyroidism-an ancillary study within two randomized placebo controlled trials. Age Ageing..

[30] Hanke L., Poeten P., Spanke L., Britz S., Diel P. (2023). The Influence of Levothyroxine on Body Composition and Physical Performance in Subclinical Hypothyroidism. Horm. Metab. Res..

[31] Chen J., Wei L., Zhu X., Xu W., Zou Y., Qi X., Fang J., Wang X., Shi X., Sheng Y. (2023). A More Practical Indicator for Evaluating the Relationship Between Sarcopenia and Thyroid Hormone in the Euthyroid Elderly Compared with FT3. Clin. Interv. Aging..

[32] Ahn S.H., Seo D.H., Cho Y., Jung M., Kim S.H., Hong S. (2020). Different Relationships Between Thyrotropin and Muscle Strength According to Sex and Age in Euthyroid Koreans (The 6th Korea National Health and Nutritional Examination Survey 2014–2015). Thyroid.

[33] Greco F., Moulton C., Antinozzi C., Lista M., Di Luigi L., Dimauro I., Sgrò P. (2023). Relationship between Euthyroidism and Muscle Mass and Strength: A Systematic Review. Int. J. Sports. Med..

[34] Wang Z., Wu P., Yang J., Jiang Y., Wang J., Lin C. (2024). Serum FT3/FT4, but not TSH is associated with handgrip strength in euthyroid U.S. population: Evidence from NHANES. Front. Endocrinol..

[35] Chatzivasileiou P., Armeni E., Chedraui P., Kontou L., Augoulea A., Palaiologou A., Kaparos G., Panoulis K., Alexandrou A., Vlachos N. (2024). Postmenopausal women with higher TSH values within the normal range present improved handgrip strength: A pilot study. Gynecol. Endocrinol..

[36] Huret C., Ferrayé L., David A., Mohamed M., Valentin N., Charlotte F., Savignac M., Goodhardt M., Guéry J.C., Rougeulle C. (2024). Altered X-chromosome inactivation predisposes to autoimmunity. Sci. Adv..

[37] Miquel C.H., Faz-Lopez B., Guéry J.C. (2023). Influence of X chromosome in sex-biased autoimmune diseases. J. Autoimmun..

[38] Migliore L., Nicolì V., Stoccoro A. (2021). Gender Specific Differences in Disease Susceptibility: The Role of Epigenetics. Biomedicines.

[39] Cho W.K. (2024). Lifelong medical challenges and immunogenetics of Turner syndrome. Clin. Exp. Pediatr..

[40] Kanakatti Shankar R. (2020). Immunological Profile and Autoimmunity in Turner Syndrome. Horm. Res. Paediatr..

[41] Gawlik A.M., Berdej-Szczot E., Blat D., Klekotka R., Gawlik T., Blaszczyk E., Hankus M., Malecka-Tendera E. (2018). Immunological Profile and Predisposition to Autoimmunity in Girls with Turner Syndrome. Front. Endocrinol..

[42] Su M.A., Stenerson M., Liu W., Putnam A., Conte F., Bluestone J.A., Anderson M.S. (2009). The role of X-linked FOXP3 in the autoimmune susceptibility of Turner Syndrome patients. Clin. Immunol..

[43] Calcaterra V., Klersy C., Muratori T., Caramagna C., Brizzi V., Albertini R., Larizza D. (2011). Thyroid ultra-sound in patients with Turner syndrome: Influence of clinical and auxological parameters. J. Endocrinol. Invest..

[44] Mitsibounas D.N., Kosmaidou Z., Kontoleon P., Deligeoroglou E., Liberatou E. (1997). Hyperlipidemia and presence of thyroid autoantibodies in girls with Turner’s syndrome or mosaic variance. J. Pediatr. Adolesc. Gynecol..

[45] Viuff M.H., Stochholm K., Juul S., Gravholt C.H. (2022). Disorders of the eye, ear, skin, and nervous system in women with Turner syndrome—A nationwide cohort study. Eur. J. Hum. Genet..

[46] Fiot E., Alauze B., Donadille B., Samara-Boustani D., Houang M., De Filippo G., Bachelot A., Delcour C., Beyler C., Bois E. (2022). Turner syndrome: French National Diagnosis and Care Protocol (NDCP.; National Diagnosis and Care Protocol). Orphanet. J. Rare Dis..

[47] Witkowska-Sędek E., Kucharska A.M., Rumińska M., Paluchowska M., Pyrżak B. (2021). Decreased Thyroxine Levels during rhGH Therapy in Children with Growth Hormone Deficiency. J. Clin. Med..

[48] Song Y., Yang H., Wang L., Gong F., Pan H., Zhu H. (2021). Association of thyroid autoimmunity and the response to recombinant human growth hormone in Turner syndrome. J. Pediatr. Endocrinol. Metab..

[49] Aversa T., Messina M.F., Mazzanti L., Salerno M., Mussa A., Faienza M.F., Scarano E., De Luca F., Wasniewska M. (2015). The association with Turner syndrome significantly affects the course of Hashimoto’s thyroiditis in children, irrespective of karyotype. Endocrine.

[50] Kim B.J., Lee S.H., Isales C.M., Hamrick M.W., Kwak M.K., Koh J.M. (2018). Association of Serum TSH with Handgrip Strength in Community-Dwelling Euthyroid Elderly. J. Clin. Endocrinol. Metab..

